# Cotton Cord Coated with Cyclodextrin Polymers for Paraquat Removal from Water

**DOI:** 10.3390/polym14112199

**Published:** 2022-05-28

**Authors:** Ekkachai Martwong, Nathapong Sukhawipat, Jatupol Junthip

**Affiliations:** 1 Division of Science (Chemistry), Faculty of Science and Technology, Rajamangala University of Technology Suvarnabhumi, Phra Nakhon Si Ayutthaya 13000, Thailand; ekkachai.m@rmutsb.ac.th; 2 Division of Polymer Engineering Technology, Department of Mechanical Engineering Technology, College of Industrial Technology, King Mongkut’s University of Technology North Bangkok, Bangkok 10800, Thailand; nathapong.s@cit.kmutnb.ac.th; 3 Faculty of Science and Technology, Nakhon Ratchasima Rajabhat University, Nakhon Ratchasima 30000, Thailand

**Keywords:** paraquat, adsorption, cotton cord, cyclodextrin, 1,2,3,4-butanetetracarboxylic acid, poly (vinyl alcohol), water pollution, pseudo-second order, Langmuir isotherm

## Abstract

The contamination of hazardous agrochemical substances in water caused essential trouble for humans and the environment. The functional textile was used as an effective adsorbent for paraquat removal from an aqueous solution. The coating of anionic cyclodextrin polymer, issued from the cross-linking between 1,2,3,4-butanetetracarboxylic acid and β−cyclodextrin in the presence of poly (vinyl alcohol), on the cotton cord, was firstly investigated. Their physicochemical characteristics were also characterized by gravimetry, acid–base titration, ATR-FTIR, ^13^C NMR, TGA, and stereo-microscopy. The BDP5 system revealed 107.3% coating yield, 1.13 mmol/g COOH groups, and 95.1% paraquat removal for 25 mg/L of initial concentration. The pseudo-second-order model was appropriate for kinetics using 6 h of contact time. Langmuir isotherm was suitable with the maximum adsorption of 30.3 mg/g for paraquat adsorption. The weight loss was 10.7% and 7.8%, respectively, for water and 5% *v*/*v* of HCI in ethanol after 120 h of contact time. Finally, the reusability efficiency stayed at 88.9% after five regeneration.

## 1. Introduction

Water plays an important role in the ecosystem and socioeconomic development. Clean water and sanitation were one of the seventeen global sustainable development goals (SDGs) for 2030, classified as the Sustainable Development Goal 6 (SDG 6) [1]. SDG 6 guarantees the accessibility and sustainability of water and sanitation for everyone, which will improve water quality by pollution reduction [2]. Agrochemical substances were employed to enhance not only the crop yields but also protect the plants from pests. However, the drawbacks might cause water contamination; these pesticides could be accumulated in environments and be harmful to human health [3]. Paraquat (PQ) is water-soluble and categorized as a non-selective pesticide for plantation or defoliation. This pesticide has intimidated both the environment [4,5] and health [6,7,8].

To remedy the contaminated water by paraquat, the adsorption process is a green technology that has been applied for treating and preventing water pollution by using an eco-friendly and innovative adsorbent from sustainable and low-cost resources [9]. Furthermore, this method has possibly featured excellent removal efficiency for pollutants from water and obtained considerable attention in recent years because of the low investment costs, the minimal energy requirements, the ease of operation, the recyclability and selectivity of adsorbents, and the flexibility for technology transfer [10]. Various effective environment-friendly adsorbents were prepared for PQ removal such as activated carbon [11], bentonite [12], bio-based material [13,14,15], cellulose nanofiber [16], cyclodextrin polymers coated on textile [17,18], cyclodextrin nanosponges [19,20,21,22], kaolin [23], and microorganisms [24].

Cyclodextrin-based materials have been widely used for environmental applications [25,26,27,28,29,30,31,32,33,34,35,36,37,38,39,40,41,42,43,44,45,46,47] because cyclodextrin (CD) molecules posse a well-imposed cyclic structure with a hydrophilic exterior and a hydrophobic cavity which are capable of encapsulation of organic components with a suitable size into the cyclodextrin cavity by host–guest interaction to build an inclusion complex. Moreover, the appearance of charge or different functional groups on cyclodextrin and its derivatives could enrich the adsorption capacity of organic molecules. A tetrafunctional cross-linking agent (1,2,3,4-butanetetracarboxylic acid (BTCA)) was used as a bridging agent to establish the cross-linked network with cyclodextrin to form insoluble nanosponges for the adsorption of bisphenol A, radionuclides (Uranium (VI) and Europium (III)) [48], and paraquat [17,19,22]. BTCA was also used to react with cellulose [49,50], starch [51], poly (vinyl alcohol) [52,53], and wool [54]. Interestingly, poly (vinyl alcohol) is a linear water-soluble polymer with the existence of hydroxyl groups, which show an outstanding degree of swelling, biodegradability, and nontoxicity that is possible to also esterify with BTCA. The pulp fibers were modified with poly (vinyl alcohol) and BTCA to improve mechanical properties; the increase in PVOH quantity by up to 5 % *w*/*v* could enhance the wet tensile index [52]. Therefore, the manifestation of poly (vinyl alcohol) could promote the adsorption efficiency towards pollutants because the available hydroxyl functions demonstrate adsorption sites through hydrogen bonding [55].

CD polymers bridged BTCA provided various applications such as functionalized textile for paraquat removal [17], biosensor platform for interstitial fluid glucose detection [53], biofunctional textile for citronella oil delivery [54], and nanofibers for methylene blue adsorption [56]. However, CD polymers cross-linked with BTCA in the presence of PVOH have never been coated on the cotton cord and informed for the adsorption of paraquat from water. In this study, the coating of these polymers was firstly achieved by in situ polymerization through an esterification reaction between BTCA with β−CD, PVOH, and/or cellulose. Then, their physicochemical properties were investigated by various techniques. Finally, the adsorption study with various parameters (pH of a solution, adsorption dosage, time, and initial concentration of paraquat), the stability study, and the recyclability study were also conducted.

## 2. Materials and Methods

### 2.1. Materials

Poly (vinyl alcohol) with M_w_ = 89,000–98,000 g/mol and 99+% hydrolyzed (Sigma-Aldrich, Saint Louis, MO, USA), cotton cord (Taisonghuad, Bangkok, Thailand), β−cyclodextrin (Acros Organics, Geel, Belgium), 1,2,3,4-butanetetracarboxylic acid (Acros Organics, Geel, Belgium), sodium hypophosphite (Acros Organics, Geel, Belgium), and paraquat dichloride hydrate (Sigma-Aldrich, Saint Louis, MO, USA) were obtained from merchandise sources. Other analytical grade chemicals and ultrapure water were used for all experiments.

### 2.2. Preparation of Adsorbents

A cotton cord (1 cm thick) was cut off to obtain many pieces of 15 cm. Then, it was washed with hot water for 30 min, dried on aluminum foil in a hot-air oven (UF10, Memmert) at 100 °C, and weighed as the initial mass (noted m_i_). After that, it was dipped into 100 mL of a mixture comprising β−CD (10% *w*/*v*), BTCA (11.14% *w*/*v*), 3% *w*/*v* sodium hypophosphite (3% *w*/*v*), and the various quantity of PVOH (0.5, 2, or 5% *w*/*v*) under agitation (150 rpm) for 24 h at 30 °C, as described in Table 1 with the given name of each formulation. The sample was drained before laying on aluminum foil, cured in a hot-air oven for 90 min at 140 °C, and cleaned with hot water for 30 min to remove the unfunctionalized coating product before drying in a hot-air oven at 100 °C. Finally, the functionalized cotton cord was weighed as the final mass (noted m_f_). Weight gain was quantified according to this equation:

(1) $$\mathrm{Weight}\;\mathrm{gain}\;\left(\%\right)=\frac{m_{f}-m_{i}}{m_{i}}\times 100$$

where m_i_ and m_f_ stand, respectively, for cotton cord weight before and after the coating process. Experiments were performed in triplicate.

### 2.3. Characterization of Adsorbents

Different samples were characterized by appropriate techniques to identify the physicochemical properties. A Thermal Analyzer (STA 449 F3 model, NETZSCH, Waldkraiburg, Germany) was performed in an alumina pan under nitrogen with a rate of 10 °C min^−1^ to observe the degradation profile of the samples. A stereomicroscope (SMZ745T model, Nikon, Melville, NY, USA) connected with a digital camera (DS-Fi3) was used to investigate the morphology of the samples. A Fourier transform infrared spectroscopy (FTIR) spectrometer (Tensor 27 model, Bruker, Billerica, MA, USA) was manipulated in attenuated total reflection (ATR) mode, with the accumulation of 64 scans in the wavenumber range of 700–4000 cm^−1^ and the resolution of 4 cm^−1^, to identify the chemical groups of the samples. A ^13^C NMR spectrometer (Ascend 400 WB model, Bruker, Billerica, MA, USA) was operated at 100.62 MHz and 298 K, utilizing a contact time of 1.5 ms and a delay time of 8 s, to analyze the chemical groups of the samples. An electron microscope (JEOL 6010 model, Tokyo, Japan) was executed with an acceleration voltage of 15 kV to observe the physical characteristic of the samples.

The quantification of carboxylic groups on the samples was carried out by pH-metric titration. A 0.1 g of the modified cotton cord was soaked in a calcium acetate solution (2% *w*/*v*) of 50 mL. After sample isolation, the solution was titrated with a sodium hydroxide solution (0.05 M) using phenolphthalein as an indicator. The quantity of COOH groups (mmol) per gram of cord was expressed in terms of the ion exchange capacity (IEC) using the following equation:

(2) $$\mathrm{IEC}\;(\mathrm{mmol}/g)=\frac{C_{\mathrm{NaOH}\;}\times {\;V}_{\mathrm{NaOH}}}{m}$$

where V_NaOH_ and C_NaOH_ stand, respectively, for the equivalent volume (mL) and concentration (mol/L) of sodium hydroxide. The symbol m is to cord weight (g). Experiments were operated in triplicate.

### 2.4. Adsorption Study

#### 2.4.1. Preliminary Adsorption Study

The pH of a 25 mg/L of paraquat solution was modified with 0.1 M HCl and 0.1 M NaOH to obtain the various pH values (2, 3, 4, 5, 6, 7, 8, 9, and 10). The modified cord (50 mg) was added to a test tube before completing the previous solution. The adsorption process was run for 360 min (150 rpm) at 30 °C to study the effect of the pH of a solution.

Various quantities of modified cord (10, 25, 35, 50, 75, and 100 mg of BDP5) were prepared before adding a 25 mg/L of paraquat solution with optimal pH. The adsorption process was operated for 360 min (150 rpm) at 30 °C to scrutinize the effect of adsorbent quantity.

The determination of paraquat was quantified by a UV-Vis spectrophotometer (GENESYS 10S model, Thermo Scientific, Vantaa, Finland) using 257 nm. The percentage of removal was calculated using the following equation:

(3) $$\%\;\mathrm{Removal}\;=\frac{{(C}_{0}-C_{t})}{C_{0}}\times 100$$

where C_0_ and C_t_ stand, respectively, for the contaminant’s original and actual time concentration. Experiments were performed in triplicate.

The adsorption capacity (Q) was calculated using the following equation:

(4) $$\mathrm{Adsorption}\;\mathrm{capacity}\;\left(\mathrm{mg}/g\right)=\frac{{(C}_{0}-C_{t})\;\times \;V}{m}$$

where C_0_ and C_t_ stand, respectively, for the contaminant’s original and actual time concentration, V relates to solution volume, and m refers to cord mass.

#### 2.4.2. Kinetics Study

The modified cord (50 mg) was added to a test tube before completing the paraquat solution of 25 mg/L. The adsorption process was run at different times (30, 60, 120, 180, 360, and 540 min) at 30 °C, optimal pH, and 150 rpm to investigate the effect of the agitation time. The measurement of contaminants was explained in the early part. Two kinetics models were used to treat the experimental data as follows:

Pseudo-first-order model:

ln (Q_e_ − Q_t_) = ln Q_e_ − k_1_t (5)

Pseudo-second-order model:

(6) $$\frac{t}{Q_{t}}=\frac{1}{k_{2}Q_{e}^{2}}+\frac{1}{Q_{e}}t$$

where Q_t_ and Q_e_ are the doses of paraquat adsorbed (in mg/g) at the actual time and equilibrium, respectively, k_1_ (/min) and k_2_ (g/mg·min) are adsorption rate constant, and t is agitation time (min). Experiments were performed in triplicate.

The intraparticle diffusion model was applied to the experimental data to study the adsorption mechanism by the following equation.

Intraparticle diffusion model:

Q_t_ = k_3i_t^0.5^ (7)

where Q_t_ is the dose of paraquat adsorbed (in mg/g) at the actual time, k_3i_ (g/mg·min^0.5^) is adsorption rate constant, and t is agitation time (min). Experiments were performed in triplicate.

#### 2.4.3. Isotherm Study

The modified cord (50 mg) was added to a test tube before completing the paraquat solution with different concentrations (25, 50, 150, 250, and 300 mg/L). The adsorption process was run for 360 min (150 rpm) at 30 °C and optimal pH to examine the effect of the paraquat solution. The measurement of contaminants was explained in the early part. Two isotherm models were used to treat the experimental data as follows:

Langmuir isotherm:

(8) $$\frac{C_{e}}{Q_{e}}=\frac{1}{K_{L}Q_{m}}+\frac{C_{e}}{Q_{m}}$$

Freundlich isotherm:

(9) $${\ln\;Q}_{e}={\;\ln\;K}_{F}+\frac{1}{n}{\ln\;C}_{e}$$

where C_e_ is the equilibrium concentration of paraquat, Q_e_ is the dose of paraquat adsorbed (in mg/g) at equilibrium, Q_m_ is the theoretical maximum adsorption capacity (in mg/g), K_L_ is the Langmuir isotherm constant, K_F_ is the Freundlich isotherm constant, and 1/n is heterogeneity factor.

The Chi-square test was applied to the experimental data to evaluate the reasonableness of the isotherm model. The Chi-square value (χ^2^) was calculated by the following equation:

(10) $$\chi 2=\sum \frac{{{(Q}_{e,\exp}{-\;Q}_{e,\mathrm{cal}})}^{2}}{Q_{e,\mathrm{cal}}\;}$$

where Q_e,exp_ is the dose of paraquat adsorbed (in mg/g) at equilibrium determined from the experimental data, and Q_e,cal_ is the doses of paraquat adsorbed (in mg/g) at equilibrium calculated from the models.

#### 2.4.4. Stability Study

Various solvents (water and 5% *v*/*v* of HCI in ethanol) were studied for the stability of the modified cord. The modified cord (50 mg) was initially weighed and then added to a test tube before completing a 10 mL of solvent. The stability of the adsorbent was run at 30 °C and 150 rpm to inspect the effect of the solvents.

In real time, the cord was migrated before drying for 30 min at 120 °C. Finally, the sample was weighed to calculate the weight loss was estimated using the following equation:

(11) $$\mathrm{Weight}\;\mathrm{loss}\;\left(\%\right)=\frac{m_{i}-m_{d}}{m_{i}}\times 100$$

where m_i_ and m_d_ stand, respectively, for modified cord weight and degraded cord weight. Experiments were carried out in triplicate. Then, the new solvent was added to this sample, and the process was continued as previously procedure before recalculating the weight loss.

#### 2.4.5. Reusability Study

The modified cord (50 mg) was added to a test tube before completing the paraquat solution of 25 mg/L. The adsorption process was run for 360 min (150 rpm) at 30 °C and optimal pH. The measurement of contaminants was explained in the early part. The desorption process was then operated by separation of adsorbent before cleaning with 5% *v*/*v* of HCI in ethanol for 6 h of soaking. After that, the adsorbent was washed with ultrapure water for 30 min and reconditioned for sorption in posterior cycles. The reusability performance was evaluated by the removal rate of each cycle.

## 3. Results and Discussion

### 3.1. Preparation and Characterization of Adsorbents

#### 3.1.1. Physicochemical Properties of Adsorbents

Anionic cyclodextrin polymer was successfully coated on the surface of the cotton cord by in situ polymerization through an esterification reaction between β−CD and BTCA in the presence of PVOH. The anionic character appeared due to the presence of uncross-linked carboxylic groups from BTCA, which could be ionizable into carboxylate functions and could be profitable to interact with cationic molecules.

**Figure 1:**
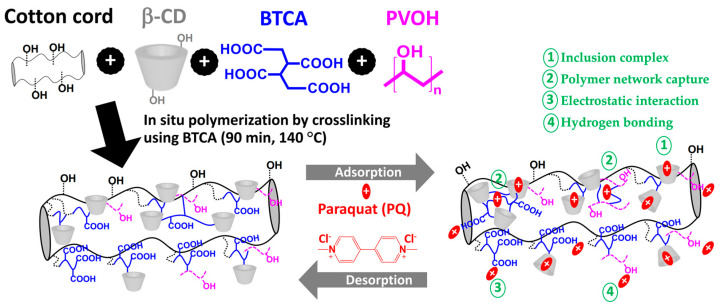
Coating reaction of modified cord and the possible adsorption mechanism with paraquat.

This cross-linking between OH functions (β−CD, PVOH, and/or cellulose) and COOH groups (BTCA) provides various cross-linked structures such as BTCA bridged with cellulose forms, BTCA bridged with β−CD forms, BTCA bridged with PVOH forms, and BTCA bridged with PVOH, β−CD, and/or cellulose. Furthermore, the availability of COOH groups from these reticulated structures could also react with PVOH, β−CD, and/or cellulose. The advantage of PVOH addition on the polymeric skeletons was to improve the adsorption site towards various pollutants by the formation of hydrogen between PVOH and pollutants. Thus, the appearance of PVOH played a supplementary role in pollutants removal from wastewater, as seen in Figure 1.

As observed in Table 2, the reference systems were also noted as BD for the system without PVOH and BP5 for the system without β−CD. The accretion of PVOH (from 0.5 to 5% *w*/*v*) increased the coating yield (from 82.2% to 107.3%). The in situ polymerization of BTCA on a cotton cord in the presence of β−CD and PVOH provides a higher coating rate because the cross-linking reaction of BTCA could be favorable with cellulose from a cotton cord, β−CD, and/or PVOH in order to establish different reticulated structures. In fact, COOH groups of BTCA could be directly esterified with OH groups of cellulose as the complementary form to solidify the coating, and the presence of the free COOH groups could also react with other substances. These results were in agreement with the previous work in which the high chance of an elevated quantity of PVOH could participate in cross-linking with pulp fibers and also improve the wet tensile index [52]. BD and BP5 systems displayed 71.9% and 48.8% of coating rates, respectively. The three-dimensional cross-linked structure of the BD system between β−CD and BTCA provided a higher coating rate compared with a linear cross-linked structure of the BP5 system between β−CD and PVOH. Therefore, the addition of PVOH increased the possibility of attachment of PVOH through the polyaddition of BTCA with PVOH, which was represented in various bridged structures as mentioned previously and enhanced the weight gain of polymer coating on the surface of the cotton cord.

However, the addition of PVOH (from 0.5 to 5% *w*/*v*) decreased the ion exchange capacity (from 1.43 mmol/g to 1.13 mmol/g) because the free COOH functions reacted with PVOH and decreased the quantity of these independent COOH groups. Thus, the ion exchange capacity was deducted with a higher quantity of PVOH.

**Figure 2:**
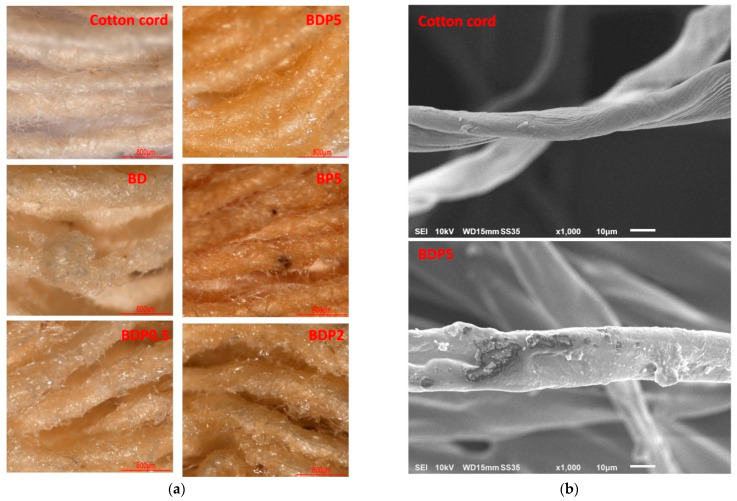
(**a**) Physical appearance of virgin cotton cord, BD, BP5, BDP0.5, BDP2, and BDP5 by stereomicroscope; (**b**) SEM images of virgin cotton cord and BDP5.

The physical appearance of the unmodified and modified cotton cord is illustrated in Figure 2a. The virgin cotton rope displayed a pale ivory color. The BD also showed a pale ivory color with a thin layer of cyclodextrin polymer. The BDP0.5 and BDP2 systems revealed a pale-yellow color with a polymer flake on the cord surface. The BDP5 system showed an intense-yellow color with a polymer lamina covering the outer cord space. The BP5 displayed an intense brown color on the rope surface. Therefore, the intensity of the brown color was increased with the quantity of PVOH because of the cross-linking with polycarboxylic acids at high temperatures. The SEM images of the virgin cotton and modified cotton (BDP5) are displayed in Figure 2b. The unmodified cord showed a smooth surface. However, the modified cord revealed a rough surface with a thin layer of cyclodextrin polymer and a polymer flake on the cord surface.

#### 3.1.2. TGA Analysis

**Figure 3:**
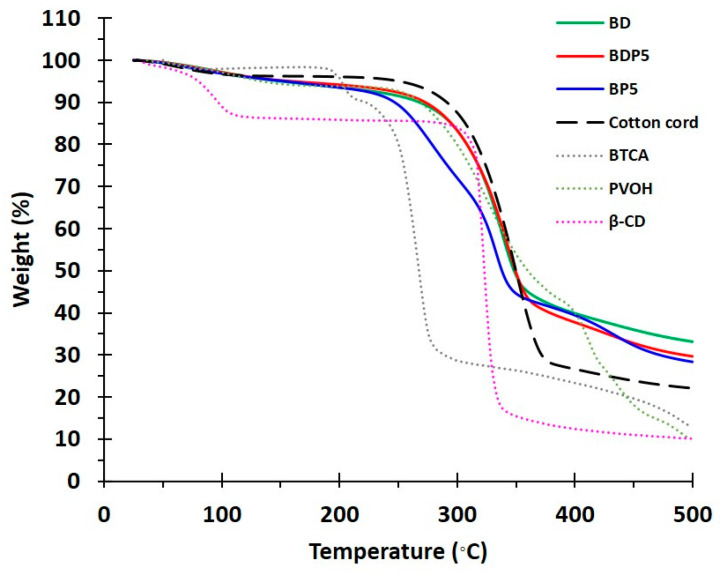
TGA thermograms of β−CD, PVOH, BTCA, cotton cord, BP5, BDP5, and BD.

The thermal stability of the modified cord was investigated by TGA, as displayed in Figure 3, for BTCA, BD, PVOH, BDP5, BP5, cotton cord, and β−CD. The loss of mass below 100 °C referred to the dehydration of samples, which was equal to 2.1%, 2.8%, 2.9%, 3.0%, 3.2%, 3.3%, and 10.7%, respectively. The thermal decomposition then started at 193 °C, 238 °C, 241 °C, 249 °C, 234 °C, 259 °C, and 296 °C, respectively. The residue above 500 °C was thermally stable with the remaining weight of 22.1%, 28.4%, 29.7%, and 33.2% for cotton cord, BP5, BDP5, and BD, respectively.

#### 3.1.3. ATR-FTIR Exploration

**Figure 4:**
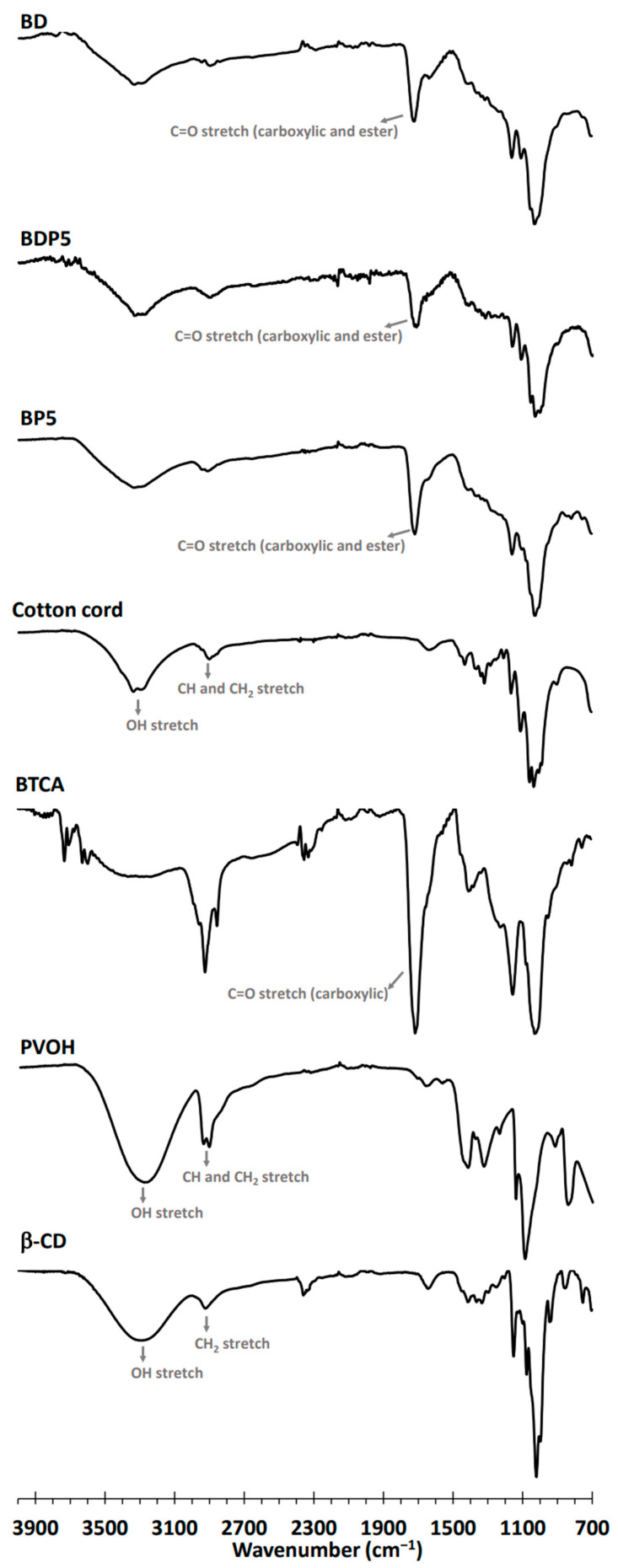
ATR-FTIR spectra of β−CD, PVOH, BTCA, cotton cord, BP5, BDP5, and BD.

As shown in Figure 4, the functional groups shown on the modified cord were characterized by ATR-FTIR. The native β−CD spectra showed particular peaks at 3288 cm^−1^, attributed to OH stretching; at 2917 cm^−1^, attributed to CH_2_ stretching; and at 1152 cm^−1^, attributed to C–O–C stretching of the glycosidic bond; they were in agreement with the data notified in the literature [57]. The PVOH spectra exhibited unique peaks at 3267 cm^−1^, attributed to OH stretching; at 2939 cm^−1^, attributed to CH stretching; at 2907 cm^−1^, attributed to CH_2_ stretching; and at 1087 cm^−1^, attributed to C–O–C stretching. The BTCA spectra revealed specific peaks at 3310 cm^−1^, attributed to OH stretching; at 2923 cm^−1^, attributed to CH stretching; at 2850 cm^−1^, attributed to CH_2_ stretching; and at 1712 cm^−1^, attributed to C=O stretching of carboxylic functions, as reported in previous works [58]. The cotton cord spectra displayed specific peaks at 3298 cm^−1^, attributed to OH stretching; and at 2896 cm^−1^, attributed to CH_2_ stretching. These results were in accordance with the data reported in the literature [59,60,61]. The characteristic peaks at 1714, 1708, and 1712 cm^−1^ for BP5, BDP5, and BD were attributed to C=O stretching of carboxylic and ester functions, which overlapped with each other. Therefore, the appearance of ester bonds verified the reaction between COOH groups from the BTCA and OH groups forming β−CD, PVOH, and/or cellulose, as informed in previous work [62].

#### 3.1.4. NMR Characterization

**Figure 5:**
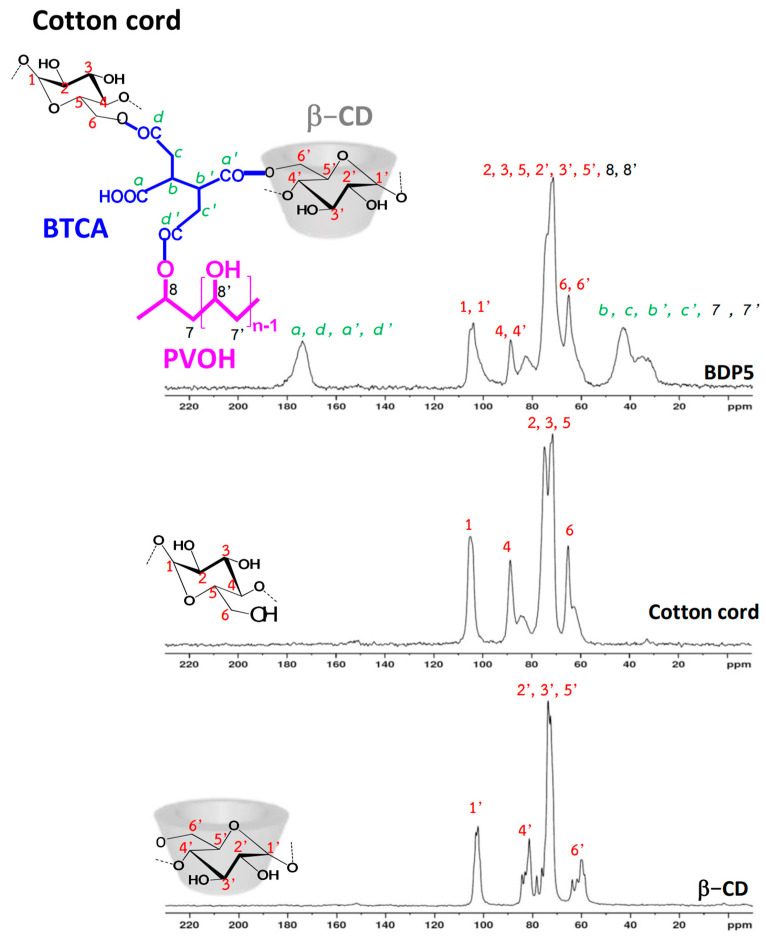
^13^C NMR spectra of β−CD, cotton cord, and BDP5.

BDP5, virgin cotton cord, and β−CD were characterized by ^13^C NMR spectroscopy to identify the chemical structure of the modified cord. Herein, the spectra of BDP5, virgin cotton cord, and β−CD are illustrated in Figure 5. The chemical shift of various reactants (spectra not shown) was described as follows: PVOH (at 44.5 ppm (for 7′) and 70.3 ppm (for 8′)) and BTCA (at 32.5 ppm (for c), 41.6 ppm (for b), 172.9 ppm (for d), and 173.5 ppm (for a)). The chemical shift of β−CD was noticed as follows: at 60.7 ppm (for 6′), 74.9 ppm (for 2′ and 5′), 78.1 ppm (for 3′), 82.9 ppm (for 4′), and 103.0 ppm (for 1′). The chemical shift of virgin cotton cord was indicated as follows: at 65.0 ppm (for 6′), 71.4 ppm (for 2′ and 5′), 74.7 ppm (for 3′), 88.7 ppm (for 4′), and 104.9 ppm (for 1′).

The ^13^C spectra of BDP5 exhibited characteristic peaks at 173.8 ppm (for a, d, a’ and d’), 105.3 ppm (for 1 and 1′), 88.8 ppm (for 4 and 4′), 71.4 ppm (for 2, 3, 5, 8, 2′, 3′, 5′, and 8′), 65.1 ppm (for 6 and 6′), and 42.6 ppm (for b, c, 7, b’, c’, and 7′). Therefore, the esterification of BTCA between β−CD, PVOH, and/or cellulose was confirmed by the few changes of chemical shift as reported in the literature [19,63,64].

### 3.2. Adsorption Study

#### 3.2.1. Preliminary Adsorption Study

The characteristic of both adsorbate and adsorbent act as a significant factor in the paraquat adsorption efficiency. Paraquat is pH-independent because of the presence of its quaternary groups. Thus, the important aspect focused on the modified cotton cord in which the COOH groups from BTCA are pH-dependent. The proposed mechanism of PQ adsorption was classified into four possibilities as shown in Figure 1: (i) creation of inclusion complex between paraquat molecules and β−CD cavity by host–guest interaction, (ii) entrapment of paraquat molecules in the polymer network, (iii) electrostatic interaction between the quaternary groups of PQ and the COOH groups of BTCA, and (iv) hydrogen bonding between hydrogen atoms of PVOH and nitrogen atoms of PQ, which was possibly reported in the literature [65].

**Figure 6:**
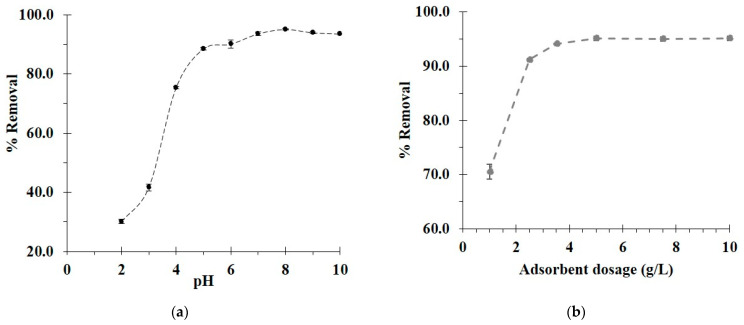
(**a**) Influence of pH on the paraquat t removal (conditions: 5 g/L of adsorbent dosage, 25 mg/L of initial concentration, 360 min of contact time, and temperature at 303 K); (**b**) influence of adsorbent dosage on the paraquat removal (conditions: optimal pH, 25 mg/L of initial concentration, 360 min of contact time, and temperature at 303 K).

The optimization of pH was first studied, as shown in Figure 6a. The BDP5 showed a low adsorption efficiency at pH 2 (30.2% of paraquat removal) because of host–guest interaction, hydrogen bonding, and cross-linked network capture. Herein, the COOH groups were deactivated because the solution pH was inferior to the pKa of BTCA (3.43, 4.58, 5.85, and 7.16). Thus, the adsorption efficiency was extremely low according to the depletion of electrostatic forces. The adsorption performance of BDP5 was gently improved with pH until reaching the maximum at a pH of 8 due to the electrostatic interaction. During this period, the COOH groups were activated to carboxylate forms which were profitable to react with cationic groups of PQ because the solution pH was superior to the pKa of BTCA. Therefore, a pH of 8 was chosen for the future experiment. Incidentally, the contamination of pesticides between the soil-bound and aqueous area was previously studied in relation to the effect of pH and ionic strength on the binding of paraquat via electrostatic interaction [66]. The adsorption of paraquat from aqueous solution by the modified cord was also based on ionic interaction when the COOH groups on the modified surface were ionizable.

The adsorbent dosage also plays an important role in the adsorption process. A 1 g/L of adsorbent dosage showed a poor removal rate of 70.6%, as seen in Figure 6b. The increase in adsorbent dosage enhanced the paraquat removal of 91.2% and 94.1% for 2 and 3.5 g/L of adsorbent dosage, respectively. The optimal adsorbent dosage attained the plateau at 5 g/L, and this quantity was selected for all experiments.

As observed in Table 2, the adsorption performances of BD, BDP0.5, BDP2, BDP5, and BP5 were 87.1%, 88.9%, 90.8%, 95.1%, and 64.5%, respectively. The BP5 is without CD, which displayed the lowest adsorption and highlighted the presence of CD to improve the adsorption performance. The increase in PVOH reduced the ion exchange capacity but outstandingly enhanced the paraquat removal according to hydrogen bonding (between PVOH and PQ) and reticulated network capture (BTCA cross-linked PVOH). These results were in accordance with the literature; the increase in PVOH in cyclodextrin polymers improved the performance of aniline extraction [67].

#### 3.2.2. Kinetics Study

**Figure 7:**
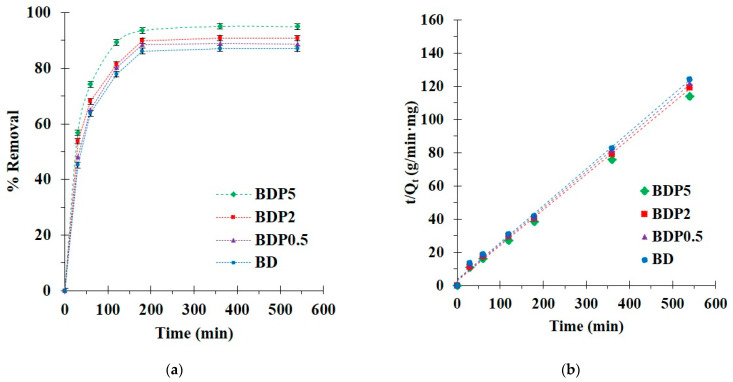
(**a**) Influence of contact time on paraquat adsorption; (**b**) pseudo-second-order kinetics of paraquat adsorption (conditions: 5 g/L of adsorbent dosage, 25 mg/L of PQ initial concentration, pH of 8, and temperature at 303 K).

The kinetics of BD, BDP0.5, BDP2, and BDP5 was performed at different contact times. The adsorption increased exceptionally for the initial 180 min before reaching the saturation of adsorption at 360 min according to the fulfilled active sites, as noticed in Figure 7a. Therefore, a contact time of 360 min was selected for the next study.

The experimental data were fitted to the pseudo-first-order and -second-order models to comprehend the adsorption process relating to an adsorption order, chemical reaction, and mass transfer. As seen in Table 3, the correlation coefficients (R^2^) were higher for the pseudo-second-order model (R^2^ = 0.9974, 0.9976, 0.9982, and 0.9986) than the pseudo-first-order model (R^2^ = 0.7966, 0.7922, 0.7998, and 0.8670 for BD, BDP0.5, BDP, and BDP5, respectively). The correlation coefficient of the pseudo-second-order model was adjacent to 1, and a straight line was obtained from this model, which proved the reasonableness of the model with experimental data, as displayed in Figure 7b. The adsorption capacity was determined by the pseudo-second-order model (Q_e,cal_ = 4.5, 4.6, 4.7, and 4.9 mg/g for BD, BDP0.5, BDP2, and BDP5, respectively).

The diffusion mechanism was explained by the intra-particle diffusion model, which was separated into two parts, as seen in Table 3. A high adsorption rate constant for the first step (k_31_) corresponded to the boundary layer diffusion; the adsorption was very fast. A low adsorption rate constant for the second step (k_32_) was attributed to the intraparticle diffusion; the adsorption was very slow. Moreover, the paraquat adsorption was a complicated process because the curve of the two parts did not pass through the origin.

#### 3.2.3. Isotherm Study

**Figure 8:**
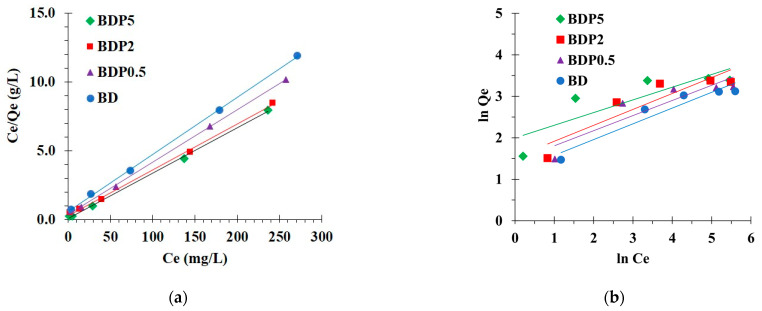
(**a**) Langmuir isotherm; (**b**) Freundlich isotherm of paraquat adsorption (conditions: 5 g/L of adsorbent dosage, 360 min of contact time, pH of 8, and temperature at 303 K).

The Langmuir (Figure 8a) and Freundlich (Figure 8b) isotherm models were applied to the experimental data at 30 °C with different initial concentrations of paraquat from 25 to 300 mg/L to assess the linearity of these models.

Isotherm parameters are displayed in Table 4. The correlation coefficient (R^2^) was essentially superior for the Langmuir isotherm model (R^2^ = 0.9997, 0.9993, 0.9980, and 0.9988) than the Freundlich isotherm model (R^2^ = 0.9162, 0.7267, 0.8253, and 0.8236) for BD, BDP0.5, BDP2, and BDP5, respectively.

A linear relationship was obtained with the Langmuir isotherm model (R^2^ closed to 1), which confirmed the suitability of the model with experimental data and described the monolayer adsorption for paraquat on the surface of the modified cord. The Chi-square values for the Langmuir model were lower than for the Freundlich isotherm model for all systems. Consequently, the Chi-square values were smaller, which confirmed the suitability of the experimental data with the Langmuir isotherm. If 0 < R_L_ (separation factor) < 1, these factors decreased with the increase in initial concentrations, which revealed a robust affinity between modified cord and paraquat.

As shown in Table 4, the maximum adsorption capacity was equal to 24.1, 26.2, 29.9, and 30.3 mg/g for BD, BDP0.5, BDP2, and BDP5, respectively. However, the removal of PQ was medium compared with other adsorbents, as seen in Table 5. Although the removal efficiency was quite medium, it could be reused many times by desorption with a suitable solvent.

#### 3.2.4. Stability Study

**Figure 9:**
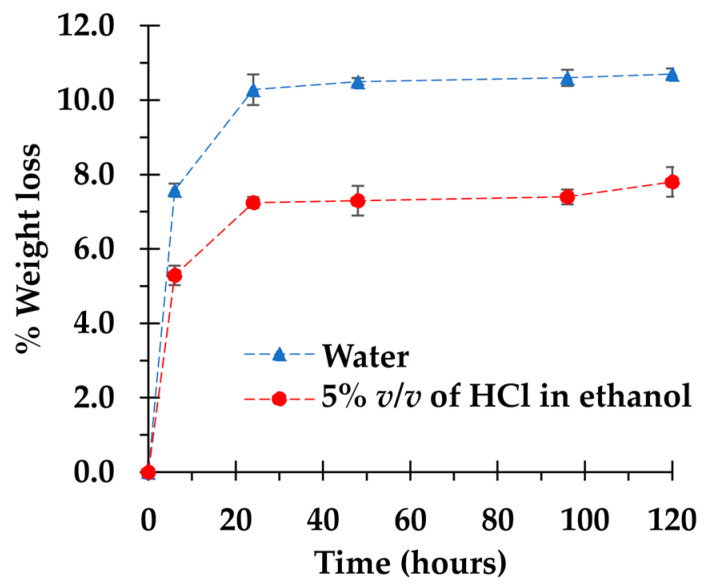
Stability of BDP5 system in water and 5% *v*/*v* of HCl in ethanol.

The stability study of adsorbent was performed in various solvents (water and 5% *v*/*v* of HCI in ethanol) to evaluate the solidity of coating performance after solvent contact during the adsorption and desorption process, as seen in Figure 9. After 6 h of contact time, the weight loss of BPD5 was 7.6% and 5.3%, respectively, for water and 5% *v*/*v* of HCI in ethanol. Then, BPD5 continued to degrade up to 10.7 and 7.8%, successively, after 24 h of contact time. After that, no degradation change was noticed after 120 h for both solvents. In fact, the anionic cyclodextrin polymer coated on the cotton cord was hydrosoluble, which made the sample decomposed easily in water by the hydrolysis of ester bonds and the formation of hydrogen bonds with water molecules. This result caused a decrease in reusability performance and was reported in the previous work [18,68]. Nevertheless, the weight loss in 5% *v*/*v* of HCI in ethanol is smaller than in water.

#### 3.2.5. Reusability Study

**Figure 10:**
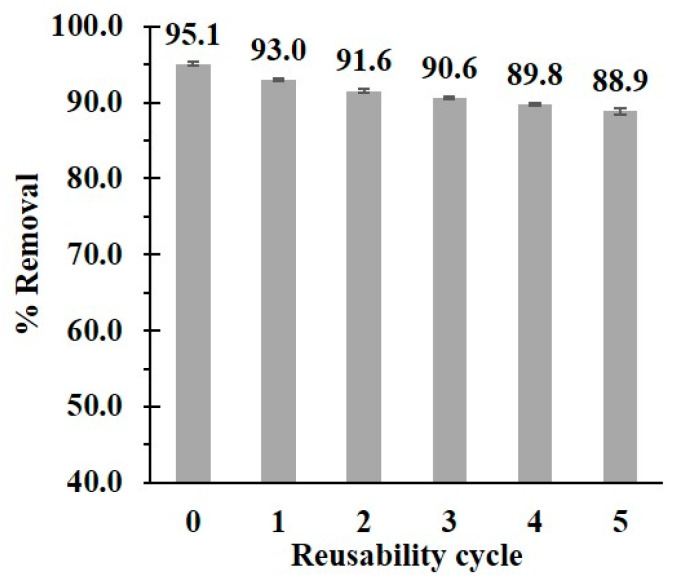
Reusability of BDP5 system for paraquat removal.

The reusability of the modified cord was investigated to assess the cost-effectiveness of the adsorption process. As shown in Figure 10, the recyclability performance decreased from 95.1% to 88.9% after five-time usage. This reduction could be explained by the degradation of polymer coating on the cotton cord surface after contact with solvents, as described in the stability study.

## 4. Conclusions

The functionalized cotton cord was accomplished by in situ polymerization between 1,2,3,4-butanetetracarboxylic acid and β−cyclodextrin in the presence of poly (vinyl alcohol) at 140 °C and 90 min. This environmentally friendly process provided 107.3% coating yield, 1.13 mmol/g COOH groups, and 95.1% paraquat removal for the BDP5 system. The physicochemical properties of these modified cords were testified by different characterization techniques. The adsorption of paraquat on the treated rope was proposed in four ways: (i) entrapment of paraquat molecules into the β−CD cavity by host–guest interaction, (ii) imprisonment of paraquat molecules in the cross-linked structure, (iii) electrostatic forces between the cationic charge of paraquat and the anionic charge of BTCA, and (iv) hydrogen bonding between nitrogen atoms of paraquat and hydrogen atoms of PVOH. Consequently, the complementary function of CD-based adsorbent came from hydrogen bonding to eradicate paraquat from water. The pseudo-second-order model and the Langmuir isotherm were suitable for kinetics (6 h of contact time) and isotherm study (the maximum adsorption of 30.3 mg/g), respectively. Eventually, the recyclability performance stood still at 88.9% after five regeneration. This efficient adsorbent could be a promising green adsorbent to defeat cationic organic pollutants from water, and this method of coating could apply on different platforms for specific purposes.

## Figures and Tables

**Table 1:** Different formulations for cotton cord coating.

Name	Composition (%*w*/*v*)		
	β−CD	BTCA	PVOH
BD	10	11.14	-
BDP0.5	10	11.14	0.5
BDP2	10	11.14	2
BDP5	10	11.14	5
BP5	-	11.14	5

**Table 2:** Physicochemical properties of cotton cord and their derivatives.

Name	% Weight Gain		IEC (mmol/g)		% Paraquat Removal	
	Mean	S.D.	Mean	S.D.	Mean	S.D.
BD	71.9	1.3	1.71	0.03	87.1	0.2
BDP0.5	82.2	0.8	1.43	0.06	88.9	0.3
BDP2	91.5	0.3	1.25	0.07	90.8	0.8
BDP5	107.3	1.9	1.13	0.05	95.1	1.1
BP5	48.8	1.5	1.89	0.04	64.5	0.7

**Table 3:** Pseudo-second-order and pseudo-first-order kinetics parameters (conditions: 5 g/L of adsorbent dosage, 25 mg/L of initial concentration, pH of 8, and temperature at 303 K).

	Q_e (exp)_	Pseudo-First Order			Pseudo-Second Order					Adsorption Mechanism	
		R^2^	Q_e (cal)_	k_1_	R^2^	Q_e (cal)_	k_2_	h	t_1/2_	k_31_	k_32_
BD	4.36	0.7966	2.3	0.0182	0.9974	4.5	0.0138	0.3	16.1	0.2925	0.0025
BDP0.5	4.45	0.7922	2.5	0.0205	0.9976	4.6	0.0147	0.3	14.9	0.2892	0.0034
BDP2	4.54	0.7998	2.2	0.0180	0.9982	4.7	0.0157	0.3	13.7	0.2494	0.0047
BDP5	4.75	0.8670	1.9	0.0164	0.9986	4.9	0.0178	0.4	11.6	0.2298	0.0076

**Table 4:** Langmuir and Freundlich isotherm parameters (conditions: 5 g/L of adsorbent dosage, 360 min of contact time, pH of 8, and temperature at 303 K).

	Q_m (exp)_	Langmuir Isotherm									Freundlich Isotherm				
		R^2^	Q_m (cal)_	K_L_	χ^2^	R_L_ for C_0_ (mg/L)					R^2^	Q_m (cal)_	K_f_	1/n	χ^2^
						25	50	150	250	300					
BD	22.7	0.9997	24.1	0.07	0.08	0.373	0.230	0.090	0.056	0.047	0.9162	10.9	3.3	0.38	13.0
BDP0.5	25.3	0.9993	26.2	0.10	0.03	0.278	0.161	0.060	0.037	0.031	0.7267	19.7	7.4	0.31	1.6
BDP2	28.5	0.9980	29.9	0.12	0.06	0.247	0.141	0.052	0.032	0.027	0.8253	16.8	4.6	0.38	8.2
BDP5	29.6	0.9988	30.3	0.36	0.01	0.101	0.053	0.018	0.011	0.009	0.8236	14.5	4.2	0.36	15.6

**Table 5:** Langmuir isotherm for paraquat removal by various adsorbents.

Adsorbent	Adsorption Dosage	Paraquat Concentration (mg/L)	Maximum Adsorption Capacity
BTCA cross-linked CD and PVOH coated on cotton rope (This work)	0.05 g in 0.01 L	25–300 mg/L	29.6 mg/g
BTCA cross-linked CD and PVOH nanosponges [22]	0.02 g in 0.01 L	25–300 mg/L	120.5 mg/g
Citric acid cross-linked CD and PVOH nanosponges [21]	0.02 g in 0.01 L	25–300 mg/L	112.2 mg/g
BTCA cross-linked CD coated on polyester textile [17]	0.02 g in 0.01 L	10–200 mg/L	26.7 mg/g
Citric acid cross-linked CD coated on polyester textile [18]	0.02 g in 0.01 L	10–200 mg/L	21.9 mg/g
Bentonite [12]	0.04 g in 0.025 L	4–24 mg/L	11.75 mg/g
Microorganisms [24]	0.005 g in 0.015 L	0–285.7 mg/L	24.4 mg/g
Bio-based material [13]	0.069 g in 0.025 L	25–85 mg/L	20.58 mg/g
Activated carbon [11]	0.01 g in 0.01 L	1.5–45 mg/L	20 mg/g

## Data Availability

The study did not report any data.
